# Fungal colonization and candidate metabolite-mediated toxicity of *Fusarium solani* KMZW-1 against adult *Bactrocera dorsalis*

**DOI:** 10.3389/fmicb.2026.1880895

**Published:** 2026-08-18

**Authors:** Huiqin Li, Zhiyi Zhao, Mingqi Wu, Chunlan Shi, Tao Zhu, Xi Gao, Chun Xiao, Mehboob Hussain, Deqiang Qin, Guoxing Wu, Junfu Yu

**Affiliations:** 1 State Key Laboratory for Conservation and Utilization of Bio-Resources in Yunnan, Yunnan Agricultural University, Kunming, China; 2 College of Plant Protection, Yunnan Agricultural University, Kunming, China; 3 College of Big Data, Yunnan Agricultural University, Kunming, China

**Keywords:** *Bactrocera dorsalis*, entomopathogenic fungi, fungal colonization, *Fusarium solani* KMZW-1, host-pathogen interaction, metabolomic profiling, podophyllotoxin, secondary metabolites

## Abstract

This study examined the interaction between *Fusarium solani* KMZW-1 and adult *Bactrocera dorsalis*, with emphasis on cuticular colonization, adult-stage pathological effects, and candidate metabolite-mediated toxicity. KMZW-1 showed *Fusarium*-like colony and conidial morphology and colonized mainly tarsal setae, joints, and recessed cuticular regions, where fungal conidia and hyphae progressively accumulated over time. Fungal treatment reduced adult survival in a concentration-dependent manner (*P* < 0.001) and lowered egg production per female, likely due to reduced male survival and fewer mating opportunities, whereas egg hatchability, larval pupation, and adult emergence of offspring were not significantly affected (*P* > 0.05). Untargeted LC-MS/MS profiling annotated 3073 metabolites, and candidate secondary metabolites were prioritized using pathway assignment, CAS-confirmed identity, normalized peak-area abundance, reported biological activity and commercial availability. Among coumarin, umbelliferone and podophyllotoxin, podophyllotoxin showed the lowest 24 h LC_50_ values against adult flies, at 24.33 μg mL^−1^ for females and 21.03 μg mL^−1^ for males. These results indicate that KMZW-1 produces a reproducible surface-colonization-associated adult-stage phenotype in *B. dorsalis*. Podophyllotoxin should be interpreted as a prioritized candidate with confirmed topical toxicity, not as a confirmed *in vivo* virulence determinant. Further work is required to confirm metabolite identity with authentic standards, quantify metabolite production during infection, determine internal invasion dynamics, and evaluate mode of action and non-target safety.

## 1 Introduction

The oriental fruit fly, *Bactrocera dorsalis* (Hendel) (Diptera: Tephritidae), is a polyphagous tephritid pest of international significance. Its larvae develop inside host fruit, which limits direct exposure to many microbial or contact agents and makes the adult stage an important interface for host-pathogen interaction studies (Clarke et al., 2005). In this context, entomopathogenic fungi provide a useful system for examining how fungal propagules attach to the insect surface, colonize external structures, and influence adult survival and reproduction.

Entomopathogenic fungi can infect insects through the cuticle and may reduce host survival or reproduction. The infection process usually begins with conidial attachment, followed by germination, hyphal growth, penetration, proliferation and, in some systems, host tissue colonization (Ortiz-Urquiza and Keyhani, 2013). SEM studies on *Metarhizium anisopliae*, *Beauveria bassiana* and *Lecanicillium lecanii* show that cuticular topography, including setae, intersegmental membranes and depressions, can influence where fungal structures accumulate (Alves et al., 2012; Fargues et al., 1994; Fernández-González et al., 2010; Santos et al., 2009). These studies provide mechanistic insight into fungal attachment and colonization behavior for examining KMZW-1 infection on adult *B. dorsalis*. Previous work has also reported fungal effects on insect survival, oviposition or development (Baker et al., 2018; Dimbi et al., 2013; Lacey et al., 2015; Song et al., 2024; Sookar et al., 2014).

Members of the Fusarium solani species complex are metabolically versatile fungi that can occupy different ecological niches. For KMZW-1, an insect-associated isolate, the relationships among cuticular colonization, adult-stage effects and fungal-associated metabolites remain insufficiently understood.

In a previous study, *F. solani* KMZW-1 was characterized at the genomic level and shown to be virulent against adult *B. dorsalis*. The estimated LC_50_ values were 5.662 log_10_ (spores⋅mL^−1^) for females and 4.486 log_10_ (spores⋅mL^−1^) for males, corresponding to 4.59 × 105 and 3.06 × 10^4^ spores⋅mL^−1^, respectively (Yu et al., 2024). However, conidial exposure alone may not fully explain all adult-stage effects. Fungal metabolites provide plausible candidates for toxicity-related follow-up studies, but their presence in culture-derived LC-MS/MS data should not be equated with *in vivo* production or causal pathogenicity without targeted validation.

Fungal secondary metabolites and compounds annotated in plant secondary metabolite biosynthetic pathways have been investigated as sources of insecticidal or growth-disrupting activity (Abdullah, 2019; Claydon, 1978; Souto et al., 2021; Suo et al., 2026). Coumarins, lignans, terpenoids and phenylpropanoids have been associated with contact toxicity, antifeedant effects or developmental disruption in different insect systems (Song et al., 2024; Suo et al., 2026; Yan et al., 2026). A recurrent weakness in metabolite-based studies, however, is that LC-MS/MS annotation is sometimes presented as functional evidence. Metabolomics reporting standards and small-molecule confidence frameworks emphasize that database-based annotations remain putative unless confirmed with authentic standards and orthogonal evidence (Schymanski et al., 2014; Sumner et al., 2007). To avoid over-interpretation, candidate selection should be separated from bioassay validation. Recent studies in insects and other tractable models also indicate that natural products and metabolite-linked pathways can alter stress, endocrine or transcriptional responses (Chen et al., 2024; Malik et al., 2024; Song et al., 2024; Yu-Hao et al., 2026; Zhu et al., 2024).

Here, we examined the interaction between *F. solani* KMZW-1 and adult *B. dorsalis* by combining adult-stage bioassays, microscopy, SEM, untargeted metabolomic profiling, and toxicity validation of selected candidate metabolites. The metabolomic data were used to define and narrow a candidate list, not to prove biological activity or *in vivo* metabolite production directly. This design allowed us to separate three distinct questions. Specifically, we investigated where KMZW-1 colonizes the adult cuticle, how exposure affects adult survival and reproductive output, and which prioritized fungal-associated compounds demonstrate adult toxicity under topical application.

## 2 Materials and methods

### 2.1 *Fusarium solani* KMZW-1 culture and *B. dorsalis* collection

Adult flesh flies, *Boettcherisca peregrina* (Diptera: Sarcophagidae), infected with a wild fungal isolate were collected from a pig farm at Yunnan Agricultural University. The isolate was identified as *F. solani* KMZW-1 by ITS annotation, with an identification value of 569/572 (99.48%) and a BLAST alignment score of 1038 (Yu et al., 2024). *B. dorsalis* populations were collected from fallen mango fruits in Kunming, China (23°36′N, 101°58′E; 460.97 m). Infested fruits were maintained on sandy soil until larval emergence and pupation. Adult flies were maintained in rearing cages at 29 °C, relative humidity (RH) ≥85%, and a photoperiod of 14:10 h (light:dark). RH was maintained using the incubator humidification system and monitored daily with a thermo-hygrometer; water was replenished as needed to maintain humidity favorable for fungal germination and infection.

### 2.2 Morphological observation of *F. solani* KMZW-1

*Fusarium solani* KMZW-1 was cultured on potato dextrose agar (PDA) medium. The sterilized PDA medium was poured into Petri dishes under aseptic conditions and allowed to solidify. A spore suspension or mycelial fragment was point inoculated onto the PDA surface using a sterile inoculating loop or needle and incubated at 25–28 °C for 5–7 days. Colony color, shape, margin, texture and reverse pigmentation were recorded.

For microscopic observation, a small amount of mycelium or conidia was transferred to a glass slide, stained with lactophenol cotton blue, covered with a coverslip and examined using a Keyence VHX-5000 digital microscope. Hyphal septation, hyphal width, conidiophore arrangement and conidial shape and size were observed at 100×, 400×, 800× and 1000× magnification (Leslie and Summerell, 2006).

### 2.3 Adult-stage and life-cycle effects of *F. solani* KMZW-1 on *B. dorsalis*

This experiment was designed to distinguish adult mortality from possible effects on egg production and offspring development. Two conidial concentrations were used: LC_50_ and 2 × LC_50_. The untreated CK group, in which both female and male adults were maintained under the same environmental and pairing conditions without fungal exposure, served as the negative control for the life-cycle assay. Because preliminary virulence assays indicated higher susceptibility in males than in females, the two sexes were treated and maintained separately before pairing. The grouping is shown in Table 1. The LC_50_ values used for grouping were taken from previous KMZW-1 virulence assays against adult *B. dorsalis* (Yu et al., 2024).

**TABLE 1:** Experimental grouping of female and male *B. dorsalis* adults in the life-cycle assay.

Group	Concentration	Female adults	Male adults
CK	Control	Untreated	Untreated
G2	LC_50_	Treated	Untreated
G3	LC_50_	Untreated	Treated
G4	LC_50_	Treated	Treated
G5	2 × LC_50_	Treated	Untreated
G6	2 × LC_50_	Untreated	Treated
G7	2 × LC_50_	Treated	Treated

For each treatment, 100 2-day-old females and 100 2-day-old males were used, with three biological replicates. Adults were maintained at 29 °C, RH ≥85% and 14:10 h (light:dark), with standard adult diet and water. At 8 days post-eclosion, 30 surviving females and 30 surviving males from each treatment replicate were randomly selected and paired in the same cage. Oviposition was recorded over 24 h when flies were 10 days old, and the numbers of eggs and surviving adults were recorded.

For hatchability assays, 100 eggs per replicate were randomly selected when egg production exceeded 100; when fewer than 100 eggs were obtained, all eggs from that replicate were used. All hatched larvae were reared under the same conditions until pupation, and all pupae were maintained until adult emergence. Hatching rate was calculated as the number of hatched larvae divided by the total number of eggs × 100%. Pupation rate was calculated as the number of pupae divided by the number of larvae × 100%. Emergence rate was calculated as the number of emerged adults divided by the number of pupae × 100%.

### 2.4 Scanning electron microscopy of fungal infection

Adult *B. dorsalis* were inoculated with an *F. solani* KMZW-1 conidial suspension (1 × 10^8^ spores⋅mL^−1^) by immersion, with 0.1% Tween-80 as the control. Samples were collected at 1, 6, 18, 36, 48 and 72 h post-inoculation. SEM observation followed established protocols for entomopathogenic fungal infection studies (Alves et al., 2012; Fargues et al., 1994; Fernández-González et al., 2010; Santos et al., 2009).

At each time point, two to three adults were transferred into 2.0 mL centrifuge tubes and fixed overnight in 2.5% glutaraldehyde. Samples were rinsed twice with 0.1 mol L^−1^ phosphate buffer (pH 7.4) for 10 min each, dehydrated through a graded ethanol series (70%, 75%, 80%, 85%, 90%, 95% and 100%), critical-point dried with CO_2_ using a Quorum K850 critical point dryer (Quorum Technologies, Laughton, UK), mounted on stubs using conductive adhesive, sputter-coated with gold and examined by SEM. Particular attention was paid to tarsal setae, antennae, wings, abdomen, dorsal cuticle, ocellar depressions, genital segments and leg articulations.

### 2.5 Untargeted metabolomic profiling and candidate prioritization

Fungal metabolites were extracted using methanol/acetonitrile (1:1, v/v; LC-MS grade, Merck), supplemented with L-2-chlorophenylalanine (≥98%, Shanghai Aladdin Biochemical Technology Co., Ltd., Shanghai, China) at a concentration of 10 μg mL^−1^ as an internal standard. Samples were homogenized at 45 Hz for 10 min, ultrasonicated in an ice-water bath for 10 min, incubated at −20 °C for 1 h and centrifuged at 12,000 rpm and 4 °C for 15 min. The supernatant was dried under vacuum and reconstituted in acetonitrile/water (1:1, v/v). After a second centrifugation, 120 μL of supernatant was transferred to autosampler vials. Quality-control samples were prepared by pooling equal aliquots from each extract.

Metabolomic profiling was performed using a Waters Acquity I-Class PLUS UPLC system coupled to a Waters Xevo G2-XS QTOF mass spectrometer (Waters Corporation, Milford, MA, USA) (Want et al., 2010). Separation was achieved on an Acquity UPLC HSS T3 column (1.8 μm, 2.1 × 100 mm; Waters) (Wang et al., 2016). The mobile phases were 0.1% formic acid in water (A) and acetonitrile (B). Data were acquired in MSE mode using MassLynx V4.2 (Want et al., 2010), with low-energy spectra at 2 V and high-energy spectra at 10–40 V, and a scan time of 0.2 s per scan.

Raw data were processed in Progenesis QI for peak extraction, retention-time alignment, deconvolution, normalization and compound annotation. Metabolites were annotated by matching accurate mass and MS/MS information against KEGG, HMDB and LIPIDMAPS databases. Relative abundance was calculated from normalized peak areas. Consistent with metabolomics reporting recommendations, these database-based annotations were treated as putative unless verified with authentic standards (Schymanski et al., 2014; Sumner et al., 2007). Because the metabolomic analysis was designed to characterize the chemical repertoire of *F. solani* KMZW-1 rather than compare multiple treatment groups, differential metabolite discovery, PCA, OPLS-DA and VIP-based biomarker screening were not applied. Instead, candidate prioritization was based on a transparent rule set: pathway assignment to secondary-metabolite-related pathways, CAS-confirmed identity, relative abundance, previous evidence of insecticidal or bioactive properties, commercial availability, regulatory feasibility and suitability for topical adult bioassays.

### 2.6 Toxicity assay of selected candidate metabolites

Three candidate metabolites were selected for adult topical bioassays: podophyllotoxin (CAS No. 518-28-5, ≥98%), coumarin (CAS No. 91-64-5, ≥98%) and umbelliferone (CAS No. 93-35-6, ≥98%) (Shanghai Yuanye Bio-Technology Co., Ltd., Shanghai, China). Acetone (analytical grade, Yunnan Xinlanjing Chemical Industry Co., Ltd., Kunming, China) was used as solvent and solvent control. Stock solutions were protected from light and stored at 4 °C when temporary storage was required; working solutions were prepared freshly before application.

Because published data for these compounds against *B. dorsalis* adults were limited, and because larvae are protected inside fruit tissues under field conditions, a preliminary adult topical assay was first used to establish working concentration ranges. Coumarin and umbelliferone were tested at 10, 50 and 250 μg mL^−1^, and podophyllotoxin at 0.5, 5 and 50 μg mL^−1^. For each concentration, 10 females and 10 males were briefly anesthetized at −18 °C for 3 min. A 5 μL aliquot of test solution was applied to the pronotum using a micro-applicator. Mortality was recorded after 24 h.

Based on preliminary LC_50_ estimates, five concentrations were selected for each compound in the multi-concentration assay: coumarin at 50, 200, 350, 500 and 650 μg mL^−1^; umbelliferone at 50, 150, 250, 350 and 450 μg mL^−1^; and podophyllotoxin at 5, 25, 45, 65 and 85 μg mL^−1^. For each compound and concentration, 30 females and 30 males were treated. Acetone was used as the solvent control. No registered insecticide or commercial microbial product was included as a side-by-side positive reference treatment; therefore, the present LC_50_ values should be interpreted as assay-specific estimates for the tested candidate metabolites rather than as direct potency comparisons with spinosad, abamectin, commercial entomopathogenic fungal strains or other registered control products.

### 2.7 Statistical analysis

All data were recorded in Microsoft Excel 2021. One-way analysis of variance (ANOVA) was conducted in IBM SPSS Statistics 22, and mean separation was performed using LSD and Duncan’s multiple range tests when variance homogeneity assumptions were satisfied. Significance was set at α = 0.05. LC_50_ values were estimated by probit regression after log_10_ transformation of concentrations. Figures were generated using GraphPad Prism 8.3.0 and Python packages numpy v1.26.4, matplotlib v3.8.3 and statsmodels v0.14.2.

For the three compounds tested in bioassays, relative metabolomic abundance was compared descriptively with toxicity, expressed as the inverse of mean LC_50_. Because only three compounds were available for this comparison, the result was treated as a screening observation, not as a statistical metabolite-activity model.

## 3 Results

### 3.1 Morphological characteristics of *F. solani* KMZW-1

**FIGURE 1:**
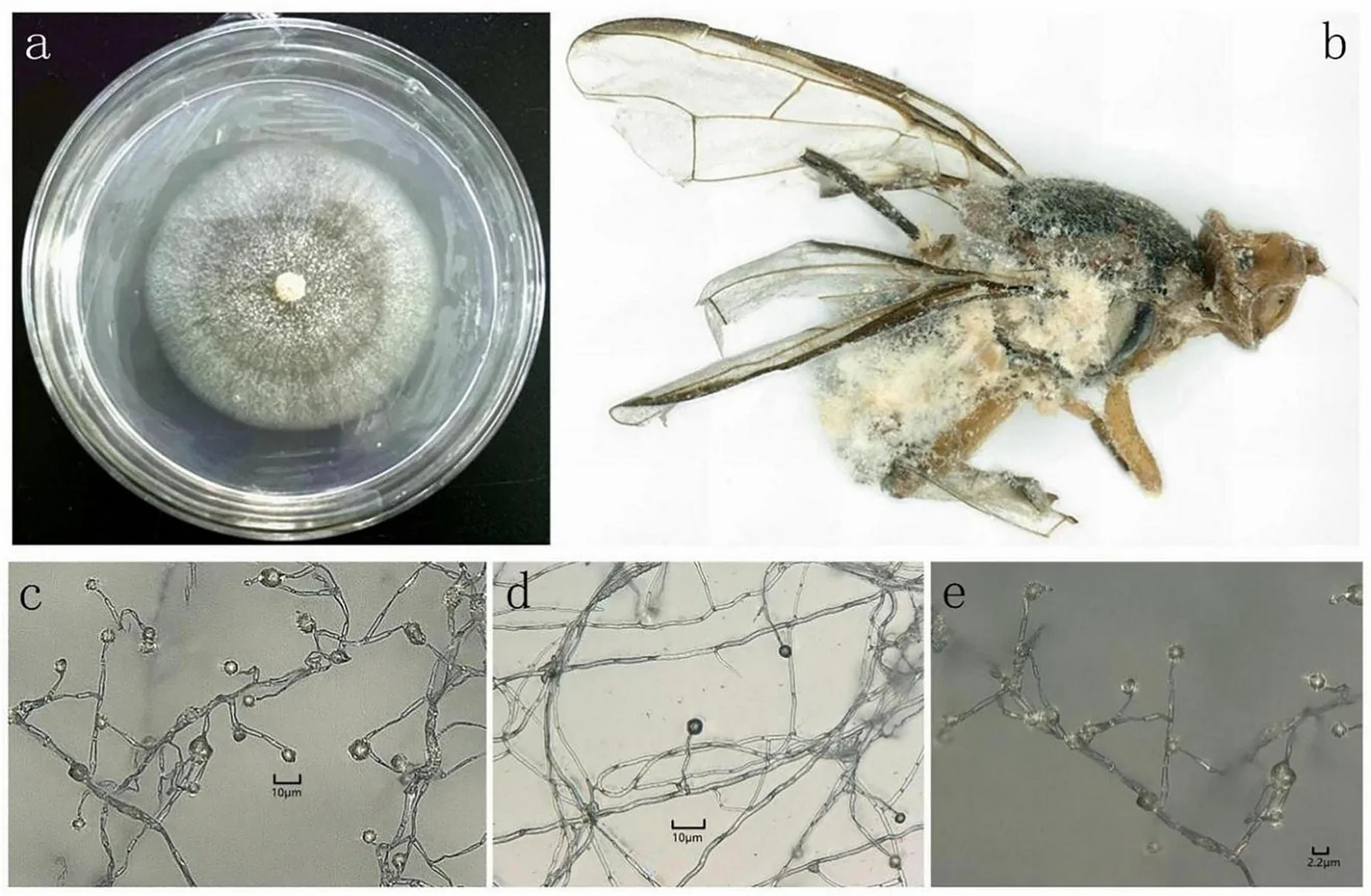
Morphological characteristics of *Fusarium solani* KMZW-1. **(a)** Colony morphology on PDA medium; **(b)** hyphal growth on adult *B. dorsalis* under 150× optical microscopy; **(c–e)** aerial conidiophores and conidia under 400×, 800× and 1000× optical microscopy, respectively.

*Fusarium solani* KMZW-1 formed circular colonies on PDA with dense, velvety white-to-yellow mycelia. The colony reverse changed from creamy yellow to light brown (Figure 1a). Under 150× optical microscopy, hyphal growth was observed on the cuticle of adult *B. dorsalis*, mainly on the thorax and abdomen (Figure 1b). At 400×, 800× and 1000× magnification, aerial hyphae were colorless and septate, with a width of 1.1–2.5 μm (Figures 1c–e). Conidiophores were solitary, opposite or verticillate, and conidia were elliptical to cylindrical, measuring 2.4–6.4 × 1.6–2.8 μm (Figures 1c–e). These observations are consistent with the identification of the isolate as *F. solani* KMZW-1.

### 3.2 Effects of *F. solani* KMZW-1 on adult survival and reproductive output

**FIGURE 2:**
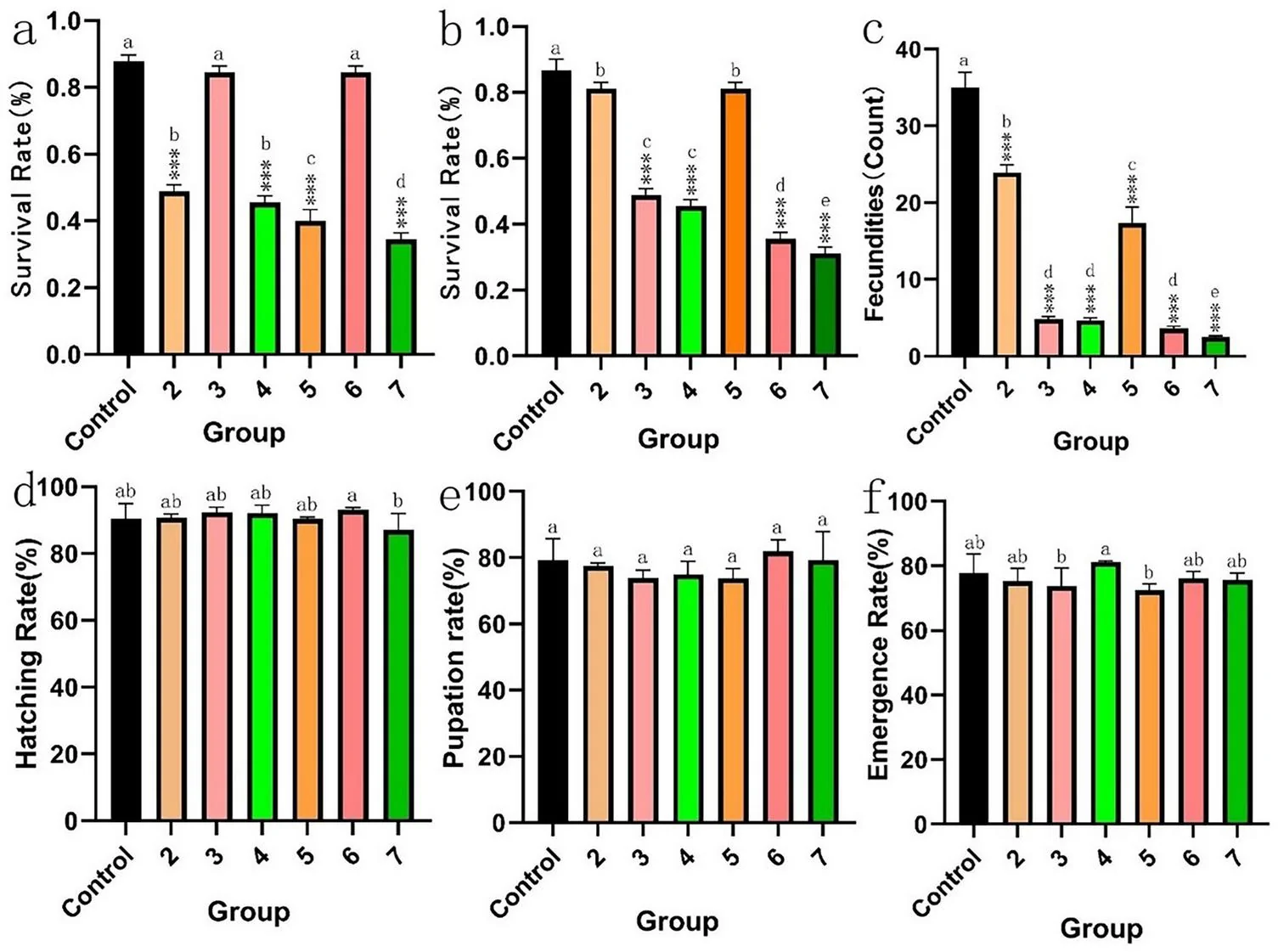
Effects of *F. solani* KMZW-1 on adult survival and reproductive output of *B. dorsalis*. **(a)** Female adult survival rate; **(b)** male adult survival rate; **(c)** eggs per female; **(d)** hatching rate; **(e)** pupation rate; **(f)** emergence rate. Different lowercase letters above bars indicate significant differences among groups according to *post hoc*-tests (*P* < 0.05). ***Indicates *P* < 0.001.

Fungal treatment reduced adult survival in a concentration-dependent manner (*P* < 0.001; Figures 2a, b). Males were more susceptible than females, and survival was lower at 2 × LC_50_ than at LC_50_. Egg production per female was also reduced (*P* < 0.001; Figure 2c). The reduction was most evident in groups with infected males, suggesting that male loss and fewer mating opportunities were major contributors to the lower egg output. Egg production at 2 × LC_50_ was lower than at LC_50_ only when both sexes were infected (*P* < 0.05).

By contrast, fungal treatment did not significantly change the offspring developmental indicators measured here. Egg hatching, larval pupation and adult emergence remained high and did not differ significantly from the control (*P* > 0.05; Figures 2d–f). Thus, under these assay conditions, KMZW-1 mainly affected adult survival and reproductive output rather than egg viability or immature development.

### 3.3 Infection process and attachment sites observed by SEM

**FIGURE 3:**
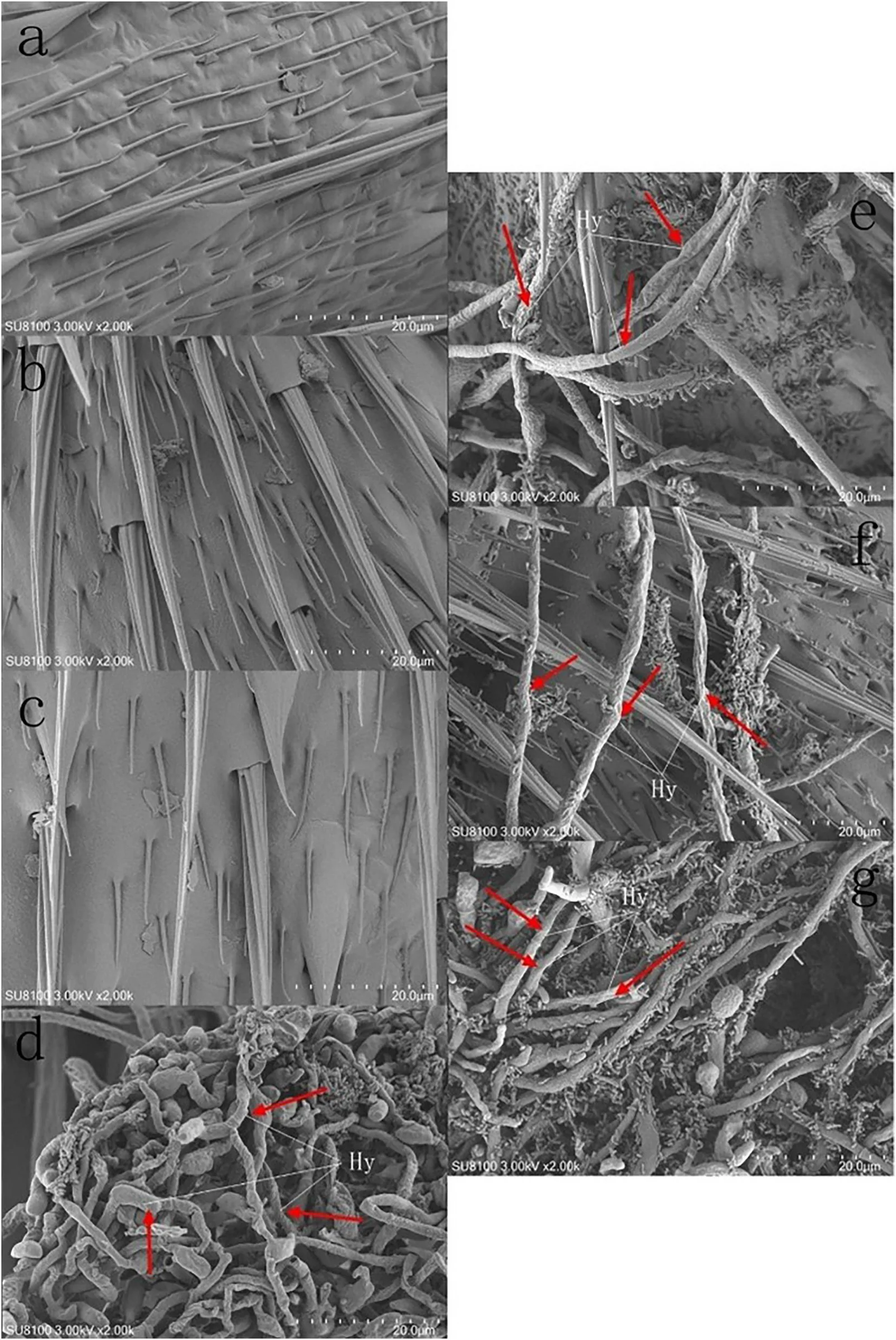
Infection progression of *F. solani* KMZW-1 on tarsal setae of *B. dorsalis* adults. **(a)** Control; **(b)** 1 h; **(c)** 6 h; **(d)** 18 h; **(e)** 36 h; **(f)** 48 h; **(g)** 72 h after inoculation. Hy = hyphae; arrows indicate fungal structures. All panels show tarsal setae observed at 2000× magnification. Scale bar = 20 μm.

SEM showed a time-dependent infection process on adult cuticle. In the untreated control, no conidia or hyphae were detected on tarsal setae (Figure 3a). At 1 h and 6 h after inoculation, obvious hyphae were not yet visible, indicating an early attachment phase (Figures 3b, c). By 18 h, hyphae were visible, with intact filaments, occasional terminal swelling and early aggregation (Figure 3d). Hyphal abundance increased at 36 h (Figure 3e). By 48 h, hyphae formed interconnected networks that partly covered the tarsal surface (Figure 3f). At 72 h, a hyphal mesh covered the observed region (Figure 3g).

**FIGURE 4:**
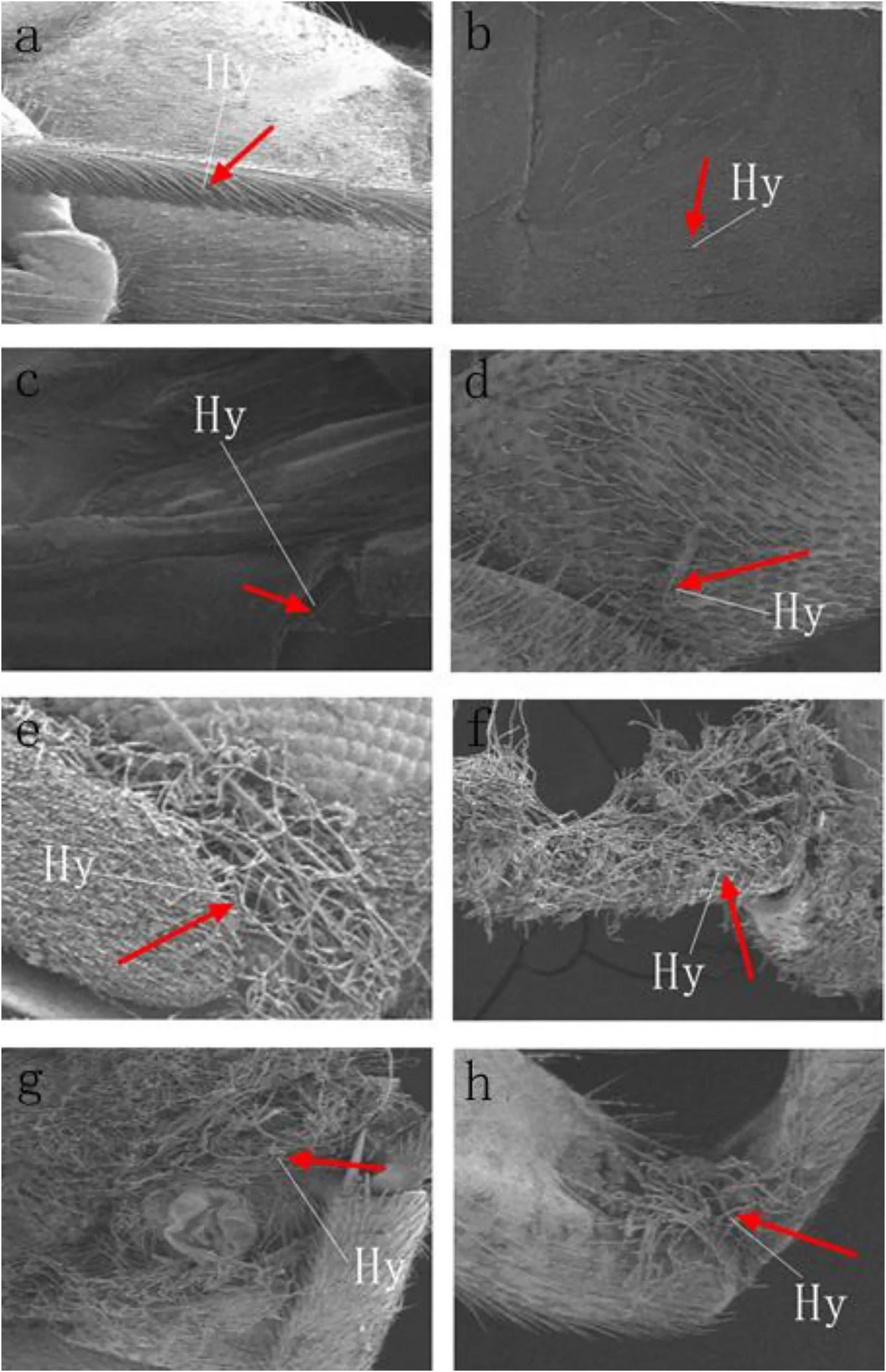
Representative cropped regions showing the distribution of *F. solani* KMZW-1 conidia and hyphae on adult *B. dorsalis*. **(a)** Antenna; **(b)** abdomen; **(c)** wing; **(d)** dorsal side; **(e)** ocellar depression; **(f)** tarsal region; **(g)** abdominal genital segment; **(h)** leg articulation. Hy = hyphae; arrows indicate fungal structures. All panels were cropped from the same whole-body SEM overview acquired at 30× magnification, in which the original scale bar was 1.00 mm. The cropped panels are intended to show the distribution of fungal structures across body regions, not to provide independent morphometric measurements.

Attachment was uneven across the insect surface. Conidia and hyphae were more frequent in recessed and setose regions, including antennae, dorsal setae, ocellar depressions, tarsal setae, abdominal genital segments and leg articulations (Figures 4a, d–h). Smoother areas, such as the abdomen and wings, had fewer attached structures (Figures 4b, c). These patterns are consistent with the view that roughness, depressions and setae help retain spores and support hyphal development.

### 3.4 Metabolite annotation and classification of *F. solani* KMZW-1

**FIGURE 5:**
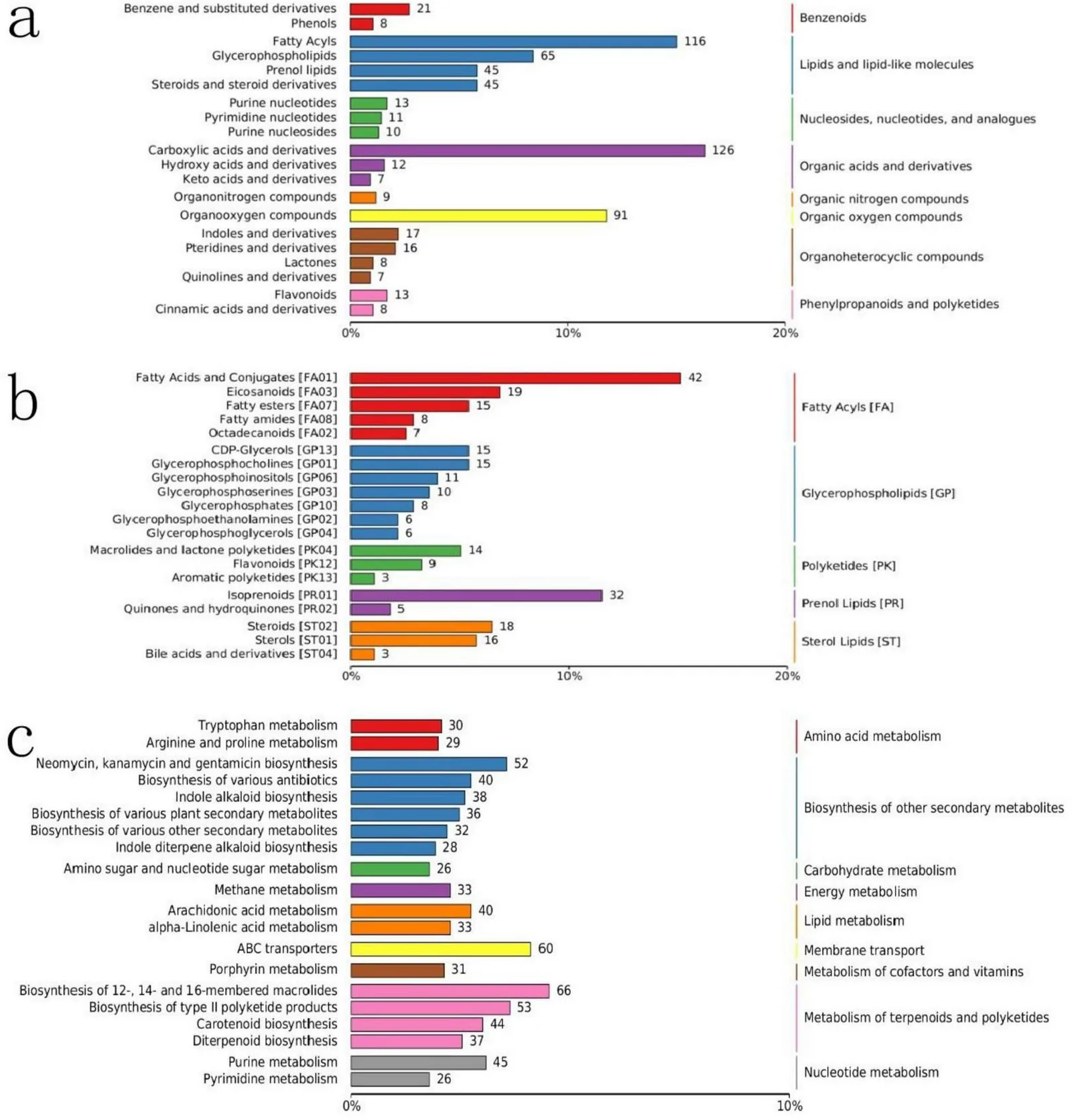
Classification and functional annotation of *F. solani* KMZW-1 metabolites. **(a)** HMDB superclass categories; **(b)** LIPIDMAPS categories; **(c)** KEGG pathway categories.

Untargeted LC-MS/MS profiling annotated 3073 metabolites using KEGG, HMDB and LIPIDMAPS. HMDB annotation assigned 1683 metabolites to eight superclass categories: benzenoids, lipids and lipid-like molecules, nucleosides/nucleotides and analogues, organic acids and derivatives, organic nitrogen compounds, organic oxygen compounds, organoheterocyclic compounds, and phenylpropanoids and polyketides (Figure 5a). Lipids and lipid-like molecules accounted for the largest HMDB category.

LIPIDMAPS annotation assigned metabolites to fatty acyls, glycerophospholipids, polyketides, prenol lipids and sterol lipids. Glycerophospholipids were the largest LIPIDMAPS class, with 156 compounds, representing 36.4% of the LIPIDMAPS-annotated metabolites (Figure 5b). KEGG annotation classified 2107 metabolites into nine major categories. Biosynthesis of other secondary metabolites was the largest category, with 252 metabolites, and included subclasses related to antibiotic biosynthesis, indole alkaloid biosynthesis, plant secondary metabolite biosynthesis and indole diterpene alkaloid biosynthesis (Figure 5c).

### 3.5 Candidate prioritization of metabolites annotated in secondary-metabolite-related pathways

**FIGURE 6:**
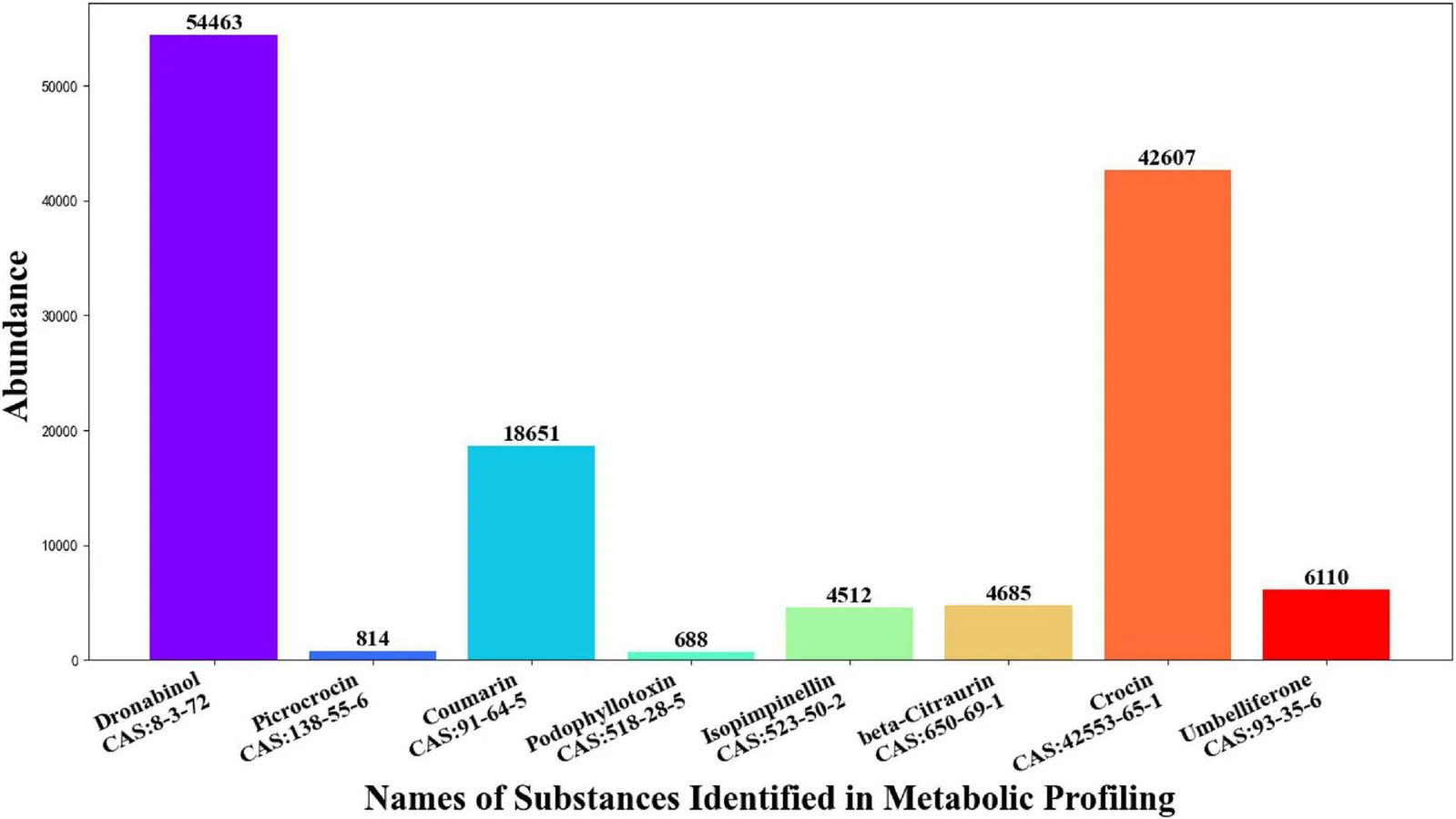
Relative abundance of representative metabolites annotated in secondary-metabolite-related pathways in *F. solani* KMZW-1 profiling. Relative abundance was calculated from normalized LC-MS peak areas. Eight metabolites were ranked; the final candidates for bioassays were selected using abundance plus feasibility and bioactivity-related criteria.

KEGG annotation identified 24 metabolites annotated to the “Biosynthesis of various plant secondary metabolites” pathway, and nine of them had CAS-confirmed identities. Eight representative metabolites were then ranked by relative abundance: dronabinol, crocin, coumarin, umbelliferone, β-citraurin, isopimpinellin, picrocrocin and podophyllotoxin (Figure 6). Selection for bioassay was not based only on peak abundance. The screening criteria also included annotation confidence, pathway membership, CAS information, published insecticidal evidence, commercial availability, cost and regulatory feasibility.

Dronabinol had the highest relative abundance but was not used because of its controlled-substance status and limited practical feasibility. β-Citraurin was not selected because insecticidal evidence and reagent availability were limited. Crocin, picrocrocin and isopimpinellin were not prioritized because of high cost or limited evidence for adult *B. dorsalis* toxicity. Coumarin, umbelliferone and podophyllotoxin met the practical criteria for the present adult topical assays and were therefore tested (Table 2).

**TABLE 2:** Multi-criteria prioritization of representative secondary-metabolite candidates for adult *B. dorsalis* bioassays.

Compound	Chemical class	CAS-confirmed	Relative abundance	Insecticidal relevance	Practical feasibility	Decision	RT (min)
Dronabinol	Terpenoid/ cannabinoid	Yes	Highest	Not prioritized for this pest	Regulatory restriction	Excluded	9.651
Crocin	Apocarotenoid glycoside	Yes	High	Limited adult-fly evidence	High cost	Excluded	2.284
Coumarin	Phenylpropanoid/ lactone	Yes	18 651.95	Reported insect bioactivity	Available, low cost	Selected	2.322
Umbelliferone	Hydroxycoumarin	Yes	6 110.77	Reported insect bioactivity	Available	Selected	2.315
β-Citraurin	Apocarotenoid	Yes	Low-to-moderate	Limited evidence	Limited supply	Excluded	7.617
Isopimpinellin	Furanocoumarin	Yes	Moderate	Potential bioactivity	High cost	Excluded	5.059
Picrocrocin	Monoterpene glycoside	Yes	Moderate	Limited insecticidal evidence	High cost	Excluded	2.165
Podophyllotoxin	Aryltetralin lignan	Yes	688.47	Reported insecticidal relevance	Available	Selected	2.493

### 3.6 Preliminary insecticidal activity assays

**FIGURE 7:**
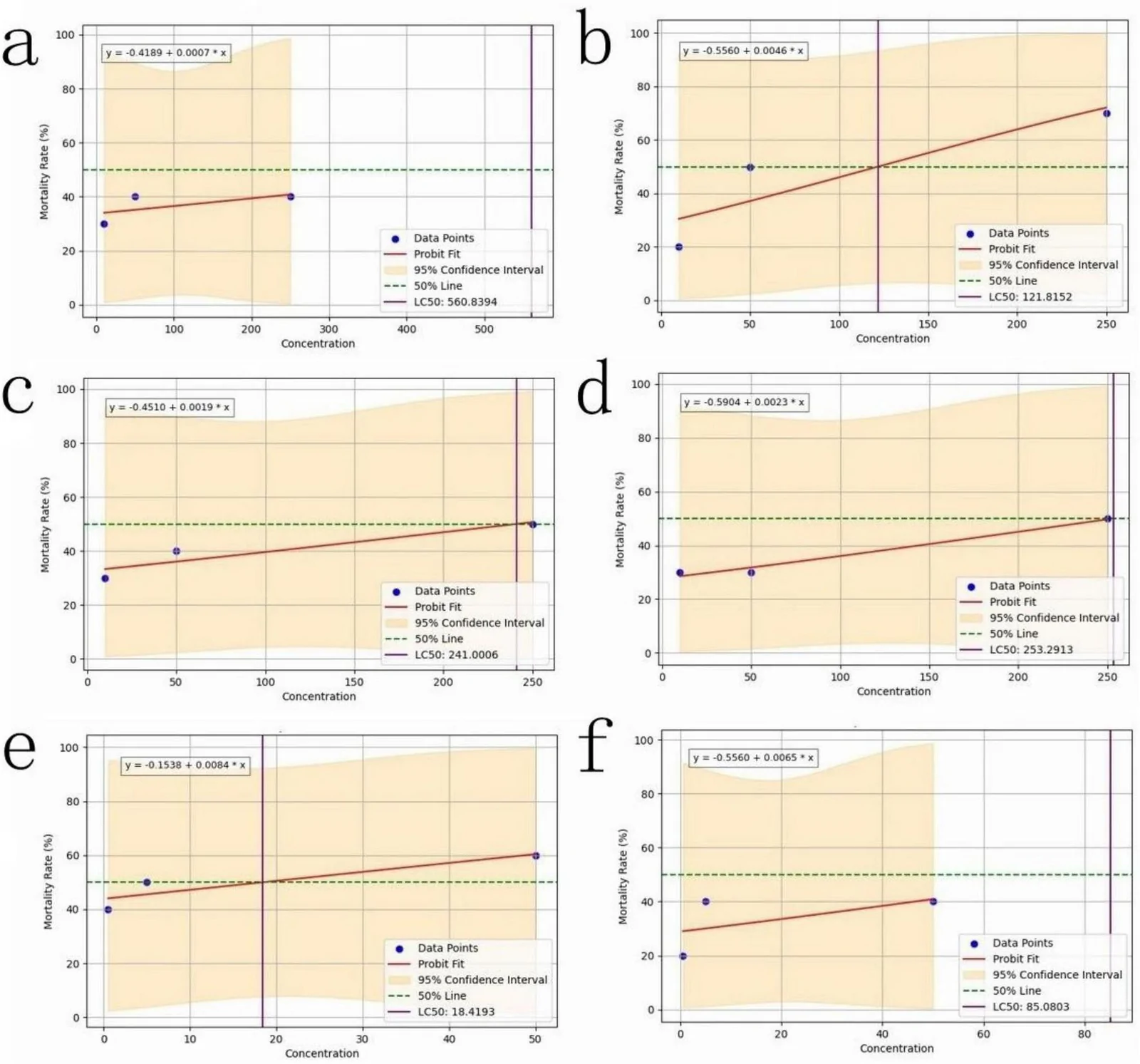
Preliminary LC_50_ estimates of coumarin, umbelliferone and podophyllotoxin against adult *B. dorsalis*. **(a, b)** Coumarin against females and males; **(c, d)** umbelliferone against females and males; **(e, f)** podophyllotoxin against females and males.

In preliminary topical assays, coumarin, umbelliferone and podophyllotoxin showed different activity patterns against adult *B. dorsalis* (Figure 7). Coumarin had estimated LC_50_ values of 560.84 μg mL^−1^ for females and 121.82 μg mL^−1^ for males. Umbelliferone had similar preliminary estimates between sexes, with LC_50_ values of 241.00 μg mL^−1^ for females and 253.29 μg mL^−1^ for males. Podophyllotoxin had LC_50_ estimates of 18.42 μg mL^−1^ for females and 85.08 μg mL^−1^ for males. These preliminary values were used to set the concentration ranges for the multi-concentration assays.

### 3.7 Multi-concentration insecticidal activity of the three candidate compounds

**FIGURE 8:**
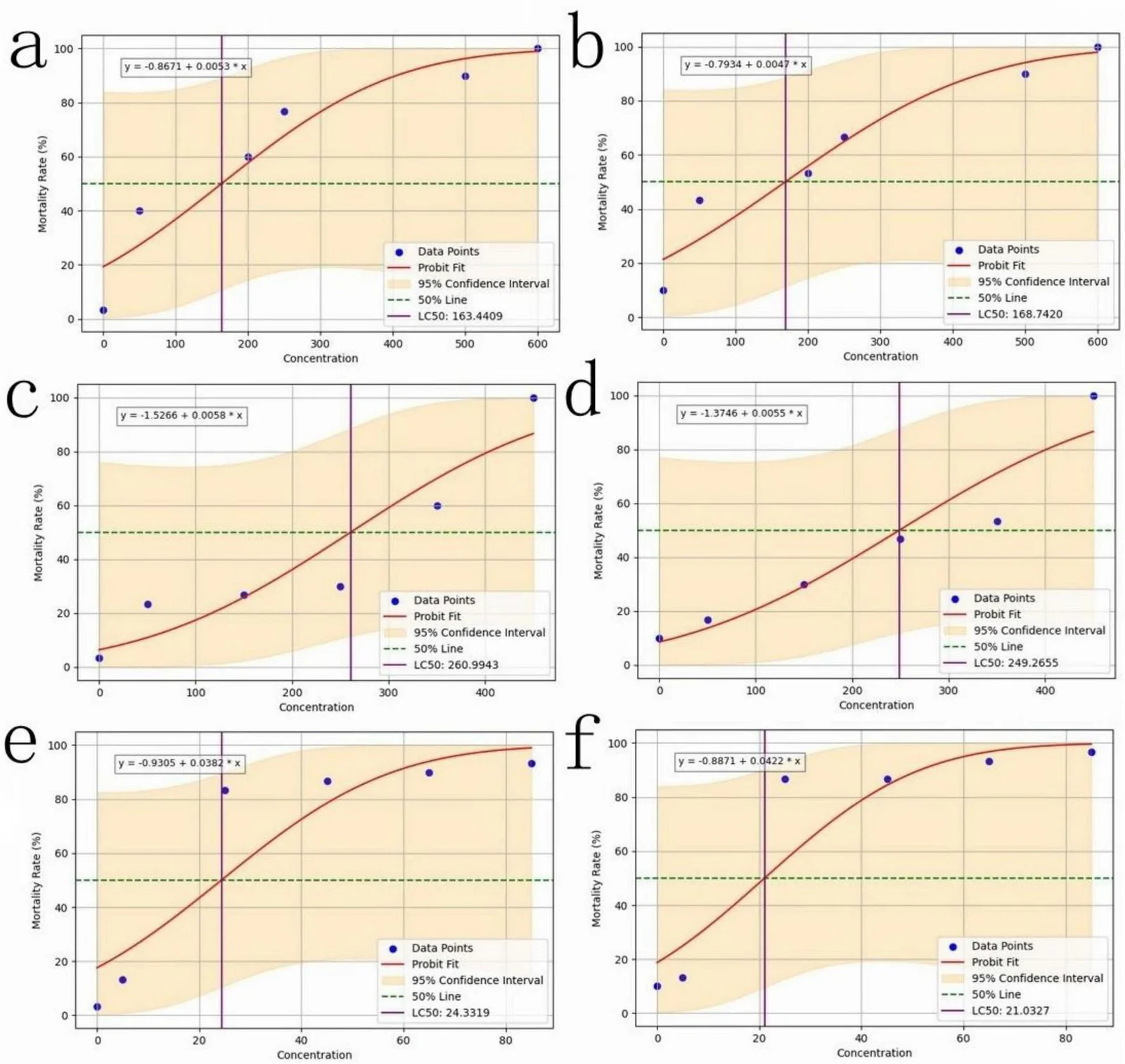
Multi-concentration topical toxicity assays of coumarin, umbelliferone and podophyllotoxin against adult *B. dorsalis*. **(a, b)** Coumarin against females and males; **(c, d)** umbelliferone against females and males; **(e, f)** podophyllotoxin against females and males.

In the multi-concentration assays, coumarin showed similar toxicity toward female and male adults, with LC_50_ values of 163.44 μg mL^−1^ and 168.74 μg mL^−1^, respectively (Figures 8a, b). The fitted regression equations were y = −0.8671 + 0.0053x for females and y = −0.7934 + 0.0047x for males. Umbelliferone was less active, with LC_50_ values of 260.99 μg mL^−1^ for females and 249.27 μg mL^−1^ for males, and regression equations of y = −1.5266 + 0.0058x and y = −1.3746 + 0.0055x, respectively (Figures 8c, d).

Podophyllotoxin had the lowest LC_50_ values among the tested compounds: 24.33 μg mL^−1^ for females and 21.03 μg mL^−1^ for males. The corresponding regression equations were y = −0.9305 + 0.0382x and y = −0.8871 + 0.0422x (Figures 8e, f). Based on mean LC_50_ values, the toxicity ranking was podophyllotoxin > coumarin > umbelliferone. A descriptive comparison between metabolomic abundance and toxicity did not show a positive abundance-toxicity relationship among the three compounds. Relative abundance alone was therefore not sufficient for candidate selection (Table 3).

**TABLE 3:** Relative abundance and 24 h topical toxicity of the three validated candidate compounds against adult *B. dorsalis*.

Compound	Relative abundance	Female LC_50_ (μg⋅mL^−1^)	Male LC_50_ (μg⋅mL^−1^)	Mean LC_50_ (μg⋅mL^−1^)	Potency rank
Coumarin	18 651.95	163.44	168.74	166.09	2
Umbelliferone	6 110.77	260.99	249.27	255.13	3
Podophyllotoxin	688.47	24.33	21.03	22.68	1

## 4 Discussion

This study describes two linked observations in the KMZW-1-*B. dorsalis* interaction. First, *F. solani* KMZW-1 reduced adult survival and egg output of *B. dorsalis*, but did not significantly affect hatching, pupation or adult emergence of the offspring measured in this experiment. Second, among the three selected compounds tested by topical application, podophyllotoxin had the lowest LC_50_ values. The reduction in egg production is most plausibly explained by lower male survival and fewer mating opportunities rather than by direct impairment of egg viability. Future studies incorporating a specific mating-frequency assay are recommended to explicitly confirm this behavioral mechanism. Similar pre-lethal or reproductive effects have been reported in other fungus-insect systems, including fruit flies and house flies exposed to *Metarhizium* or *Beauveria* species (Baker et al., 2018; Dimbi et al., 2013; Lacey et al., 2015; Sookar et al., 2014). The morphology of KMZW-1 was consistent with previous descriptions of the *F. solani* species complex (He et al., 2024; Muraosa et al., 2017).

The SEM observations show where colonization accumulated on the adult cuticle. KMZW-1 conidia and hyphae were concentrated on tarsal setae, joints, ocellar depressions and other rough or recessed areas. These sites likely provide surface roughness and local protection that favor spore retention. Similar cuticle-dependent infection patterns have been described for other entomopathogenic fungi (Alves et al., 2012; Fargues et al., 1994; Fernández-González et al., 2010; Guo, 2010; Zhang et al., 2024; Ortiz-Urquiza and Keyhani, 2013; Santos et al., 2009; Wang et al., 2009; Wang et al., 2005). The dense hyphal network observed at 48–72 h is consistent with sustained surface colonization. However, SEM of external surfaces cannot directly demonstrate internal penetration, nutrient extraction, tissue invasion or hemocoel colonization. Future studies using histological sectioning, fluorescence-labeled fungal strains or confocal microscopy will be needed to visualize the spatiotemporal dynamics of internal infection.

The metabolomic profile showed that KMZW-1 contains a broad set of annotated metabolites, including lipids and lipid-like molecules. Glycerophospholipids were abundant in the LIPIDMAPS classification. This observation should not be interpreted as direct evidence that these compounds mediate infection. It does, however, point to a testable question for future work: whether fungal lipid metabolism affects conidial surface properties, adhesion to the insect epicuticle or early colonization. As with microbial enzyme-domain annotations, broad biochemical annotation requires targeted validation before it can be used for mechanistic inference (Wei et al., 2025).

The candidate-selection process should also be interpreted as prioritization rather than causal inference. The metabolomic data generated a list of annotated compounds, and the final choices combined pathway annotation, CAS-confirmed identity, normalized abundance, published bioactivity evidence, reagent availability, cost and regulatory feasibility. Consistent with metabolomics confidence frameworks, the compound identities reported here should be viewed as putative annotations unless they are confirmed by authentic standards and targeted analysis (Schymanski et al., 2014; Sumner et al., 2007). The three-compound comparison showed that peak abundance did not predict topical toxicity: podophyllotoxin was less abundant than coumarin and umbelliferone but was more active in the bioassay. This supports the use of multiple screening criteria rather than abundance alone, and highlights the need to explore structure-activity relationships (SAR) to better understand the differential toxicity among these fungal-associated compounds.

Podophyllotoxin had the lowest LC_50_ values in the adult topical assay. This result is consistent with previous reports that podophyllotoxin derivatives can have insecticidal activity and may interfere with developmental or signaling processes (Che et al., 2013, 2019; Di et al., 2007; Liu et al., 2011; Miyata et al., 2019; Sun et al., 2017, 2022; Tian et al., 2024; Yang et al., 2018; Zhang et al., 2021; Zhi et al., 2017). Coumarin and umbelliferone were less toxic under the same assay format. Coumarin-related compounds have been linked to detoxification enzymes, acetylcholinesterase or glycometabolism in insects (Bandyopadhyay et al., 2024; Li et al., 2024; Miyashita et al., 2024; Roca-Acevedo et al., 2015; Sharma et al., 2006; Vargas-Soto et al., 2017; Wang et al., 2012; Xia et al., 2023), while umbelliferone activity can vary by species and detoxification capacity (Brattsten, 1987; Sunita et al., 2013; Wang et al., 2012; Xiaorong and Taiping, 2008). These comparisons suggest possible mechanistic directions, but they do not identify the target sites in *B. dorsalis*.

The podophyllotoxin result should be treated as candidate toxicity evidence, not as proof that podophyllotoxin is a KMZW-1 virulence determinant *in vivo*. Podophyllotoxin is a cytotoxic lignan with known mammalian toxicological concerns (Kong et al., 2023; Ma et al., 2024; Jin et al., 2001). For a host-interaction interpretation, the next question is whether this compound is produced during fungal colonization of the insect and whether its abundance correlates with host mortality. Non-target safety evaluation should include pollinators, parasitoids, predators and other beneficial arthropods, because entomopathogenic fungi and fungal-associated metabolites can interact with multitrophic natural-enemy systems (Hajek and Butler, 2000; Roy and Pell, 2000). Recent work on bee-safe nanoinsecticide design and eco-compatible plant-derived biopesticide discovery further reinforces the need to evaluate selectivity and beneficial-insect safety (Suo et al., 2026; Yan et al., 2026). Safety discussion should also distinguish the selected candidate compounds from classical fungal mycotoxin hazards (Bennett and Klich, 2003), while taking into account known pharmacological and toxicological information for coumarins, umbelliferone and related compounds (Guo et al., 2020; Kong et al., 2023; Liliang and Wei, 2024; Lin et al., 2023; Ma et al., 2024; Mengyuan, 2022; Min et al., 2019; Murayama and Yamazaki, 2021; Pitaro et al., 2022; Jin et al., 2001).

Several limitations should be considered when interpreting the bioassay results. First, no side-by-side positive reference treatment, such as a registered insecticide or a commercial entomopathogenic fungal strain, was included in the present study. The LC_50_ values reported here should therefore be interpreted as assay-specific estimates for comparing the tested candidate metabolites under the same laboratory topical application protocol, rather than as direct potency comparisons with registered insecticides, microbial products or field-level control agents. Second, metabolite annotations were not confirmed here with authentic standards, and production of the prioritized metabolites during insect colonization was not quantified. Third, the SEM data describe external colonization but do not resolve internal invasion. Future work should include standard-based targeted LC-MS/MS analysis using infection-stage insect samples, histological or fluorescence-based localization of fungal invasion, mode-of-action assays, and exposure routes more closely related to fungal-host interaction, such as residual contact, bait ingestion, fungal culture extracts or infection-stage bioassays. Finally, although brief cold anesthesia (−18 °C for 3 min) is a standard handling procedure, its potential to induce a mild cold shock effect should be controlled for in future fine-scale physiological bioassays.

Taken together, the results support a focused interpretation. KMZW-1 colonizes external structures of adult *B. dorsalis* and reduces adult survival and egg output under laboratory conditions. Podophyllotoxin was the most active compound among the three prioritized candidates, but the data do not yet establish a causal virulence role, *in vivo* production during infection, field efficacy, environmental safety or resistance-management value. These points require separate experiments.

## 5 Conclusion

*Fusarium solani* KMZW-1 showed *Fusarium*-like morphology and colonized adult *B. dorsalis* on cuticular structures, especially rough or setose regions. Under the laboratory conditions used here, fungal treatment reduced adult survival and egg production per female, with the strongest reproductive effect observed when males were infected. Egg hatching, larval pupation, and adult emergence were not significantly changed.

Untargeted metabolomic profiling was used to support candidate selection rather than to prove biological activity. Among the three tested compounds, podophyllotoxin had the lowest 24 h LC_50_ values against adult *B. dorsalis*. The data suggest that KMZW-1-associated metabolites deserve further study in the context of fungal-insect interaction. The next steps should include authentic-standard confirmation of metabolite identity, targeted quantification during infection, mode-of-action assays, non-target safety evaluation, and tests using field-relevant exposure routes.

## Data Availability

The original contributions presented in this study are included in the article/supplementary material, further inquiries can be directed to the corresponding authors.
